# p63: a crucial player in epithelial stemness regulation

**DOI:** 10.1038/s41388-023-02859-4

**Published:** 2023-10-17

**Authors:** Yanan Li, Sara Giovannini, Tingting Wang, Jiankai Fang, Peishan Li, Changshun Shao, Ying Wang, TOR centre, Massimiliano Agostini, Pierluigi Bove, Alessandro Mauriello, Giuseppe Novelli, Mauro Piacentini, Valentina Rovella, Manuel Scimeca, Giuseppe Sica, Qiang Sun, Giuseppe Tisone, Yufang Shi, Eleonora Candi, Gerry Melino, Francesca Bernassola

**Affiliations:** 1 Department of Experimental Medicine, TOR, University of Rome Tor Vergata, 00133 Rome, Italy; 2 The Third Affiliated Hospital of Soochow University, Institutes for Translational Medicine, Soochow University, Suzhou, 215000 China; 3 Shanghai Institute of Nutrition and Health, Shanghai, 200031 China; 4 Biochemistry Laboratory, Istituto Dermopatico Immacolata (IDI-IRCCS), 00100 Rome, Italy; 5 Dipartimento di Biomedicina e prevenzione, University of Rome Tor Vergata, 00133 Rome, Italy; 6 Department of Biology, University of Rome Tor Vergata, 00133 Rome, Italy; 7 Chinese Academy of Medical Science, Beijing, 100071 China

**Keywords:** Self-renewal, Cancer stem cells

## Abstract

Epithelial tissue homeostasis is closely associated with the self-renewal and differentiation behaviors of epithelial stem cells (ESCs). p63, a well-known marker of ESCs, is an indispensable factor for their biological activities during epithelial development. The diversity of p63 isoforms expressed in distinct tissues allows this transcription factor to have a wide array of effects. p63 coordinates the transcription of genes involved in cell survival, stem cell self-renewal, migration, differentiation, and epithelial-to-mesenchymal transition. Through the regulation of these biological processes, p63 contributes to, not only normal epithelial development, but also epithelium-derived cancer pathogenesis. In this review, we provide an overview of the role of p63 in epithelial stemness regulation, including self-renewal, differentiation, proliferation, and senescence. We describe the differential expression of TAp63 and ΔNp63 isoforms and their distinct functional activities in normal epithelial tissues and in epithelium-derived tumors. Furthermore, we summarize the signaling cascades modulating the TAp63 and ΔNp63 isoforms as well as their downstream pathways in stemness regulation.

## Introduction

### Structural features and expression patterns of p63

**Fig. 1:**
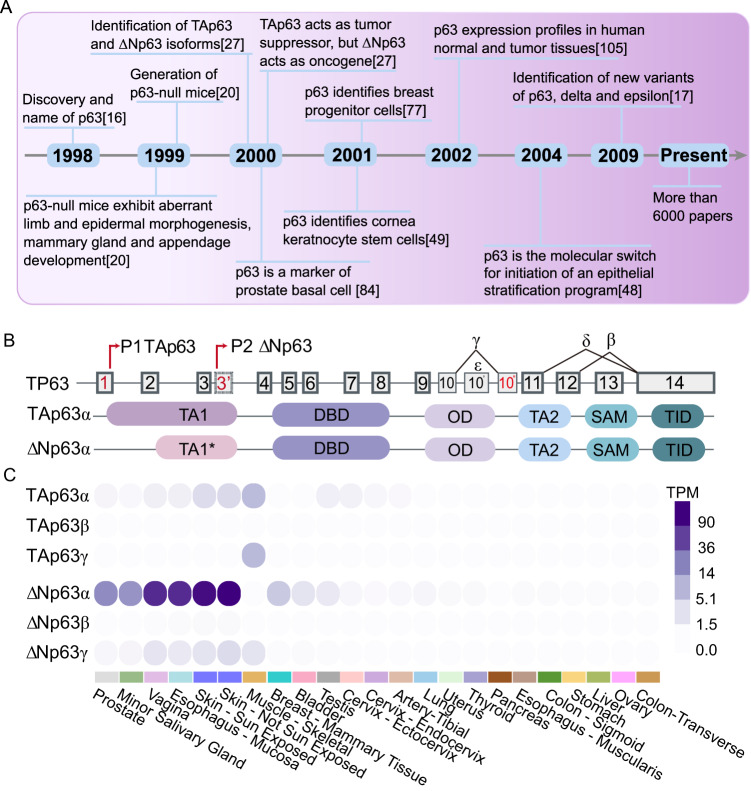
General features of p63. **A** Timeline of important discoveries in the research field of p63 in epithelial tissues. **B** Schematic representation of the alternative promoters generating the TAp63 and ΔNp63 isoforms along with structural features and domains of the p63 protein variants. p63 protein are shown: TA transactivating domain, DBD DNA-binding domain, OD oligomerization domain, SAM sterile alpha motif, TID trans-inhibitory domain. **C** The differential expression pattern of TAp63 and ΔNp63 variants (α, β, and γ) in different normal tissues (Data source, GTex portal).

p63 is the most ancient member belonging to the p53 family together with p73 [1–4] (Fig. 1A). All members are involved in essential biological functions, including cell death [5, 6], tumor suppression [7–10], and cell differentiation [11–14]. Its encoding gene TP63 generates two main classes of isoforms by the usage of alternative promoters. TAp63 transcripts are produced from the promoter upstream of exon 1 and transcribe for protein isoforms that contain an N-terminus acidic transactivation domain (transcriptional activation domain 1, TA1), homologous to the one of p53 (Fig. 1B). The usage of an alternative promoter located within the intron downstream of exon 3 generates transcripts encoding ΔNp63 isoforms that lack the canonical N-terminal TA domain 1 (TA1*). Furthermore, the TP63 gene is expressed as, at least, three alternatively spliced C-terminal isoforms (α, β, γ) [15]. The full-length p63α proteins possess a C-terminus sterile alpha motif (SAM) domain, a protein-protein interaction domain, flanked by a second transactivation domain (TA2) and a transcription inhibitory domain (TID), which can auto-inhibit the transcriptional activity of the TA isoforms. All of the isoforms share some functional domains including the DNA-binding domain (DBD) and the oligomerization domain (OD). Altogether, the TP63 gene expresses at least six different p63 protein isoforms (TAp63α, TAp63β, TAp63γ, ΔNp63α, ΔNp63β and ΔNp63γ). Minor alpha-helix changes produce profound functional differences; accordingly, these six isoforms display distinct functions. The identification of two new C-terminus variants, p63δ and ε, has expanded the isoforms number from 6 to 10 [16, 17]. Moreover, two additional N-terminal short splicing isoforms lacking exon 4 have been found in invasive breast carcinomas (d4TAp63 and ΔNp73L) [18].

The TAp63 isoforms are generally expressed at low basal levels in epithelial cells, while their expression can be induced in response to DNA damage or other cellular stresses. ΔNp63 isoforms are instead constitutively expressed in the basal compartment of the epidermis and in other epithelial tissues [19–21]. Unlike TAp63, ΔNp63 expression is downregulated in response to genotoxic stress. TAp63 binds canonical p53-responsive elements (RE), thus activating the transcription of several p53 target genes, and therefore acting as a tumor suppressor through the regulation of cell-cycle arrest and apoptosis [16, 22–26]. Conversely, the ΔNp63 isoform acts as an oncogene [27, 28]. The ΔNp63 isoforms are mainly involved in maintaining the self-renewing capacity of progenitor cells in different epithelia [29–32]. TAp63 and ΔNp63 variants are typically and differentially expressed in epithelial tissues and their appendixes [33, 34]. For example, ΔNp63 (mainly ΔNp63α) is the most predominant isoform expressed in the skin, vagina and esophageal mucosa. Furthermore, TAp63 and ΔNp63 isoforms are present during distinct stages of embryonic development [35]. During epidermal development, while the TAp63 variants are expressed in the uncommitted ectoderm surface, the ΔNp63 isoforms are transcribed only after the surface ectoderm has been committed towards a stratification program [36].

### Role of p63 in ectodermal development and epithelial homeostasis

Epithelial tissues cover external surfaces of organs and blood vessels, as well as the internal surfaces of cavities in many internal organs [37] These tissues participate in a diverse range of physiological functions, including absorbing nutrients, filtering and eliminating toxic metabolic by-products, retaining body fluids, regulating body temperature and, in the case of epidermis, the outmost layer of the skin, acting as effective barriers against pathogens [38, 39]. Based on their structure and function, the epithelium can be divided into three categories: covering epithelium, glandular epithelium, and sensory epithelium. Epithelial tissue homeostasis is preserved by ESCs located within specialized niches which are responsive to the regenerative demands of the tissue [38, 40]. p63, as an indispensable transcription factor for epithelial morphogenesis and stemness [19, 20], is highly expressed in the basal or progenitor layers of various epithelial tissues [16] (Fig. 1C). As a result, p63-deficient mice lack epidermis and other stratified epithelia [20, 34]. The mice are born alive but die shortly after birth because of severe dehydration, and manifest dramatic developmental defects, affecting particularly their limbs and skin [19]. In addition, absence of p63 results in the failure of basal cell population maintenance, suggesting an essential role of p63 in the self-renewing of epithelial stem cell [41, 42]. Besides, cancerogenesis and epithelium tissue regeneration are somewhat related process sharing similar mechanisms, both involving a p63-cascaded regulation [2, 43]. The shared mechanisms between cancerogenesis and tissue regeneration highlight the complex nature of p63-mediated processes. Understanding the intricate interplay between p63 and its downstream targets in both contexts is essential for unraveling the underlying molecular mechanisms and potentially identifying therapeutic strategies for cancer treatment and tissue regeneration.

## Role of p63 in epithelial stem cell biology

The self-renewing ability of stem cells located in the innermost basal layer is crucial for maintaining stratified epithelial tissue homeostasis, as basal cells move up and become suprabasal cells, they lose their capacity to proliferate and instead undergo a terminal differentiation program [44, 45]. Mice lacking p63 fail to develop stratified epithelia, epithelial appendages, as well as glandular tissues, indicating that p63 is required for the commitment of epithelial stem cells (ESCs) during embryonic development [29, 46–48].

### Skin

**Fig. 2:**
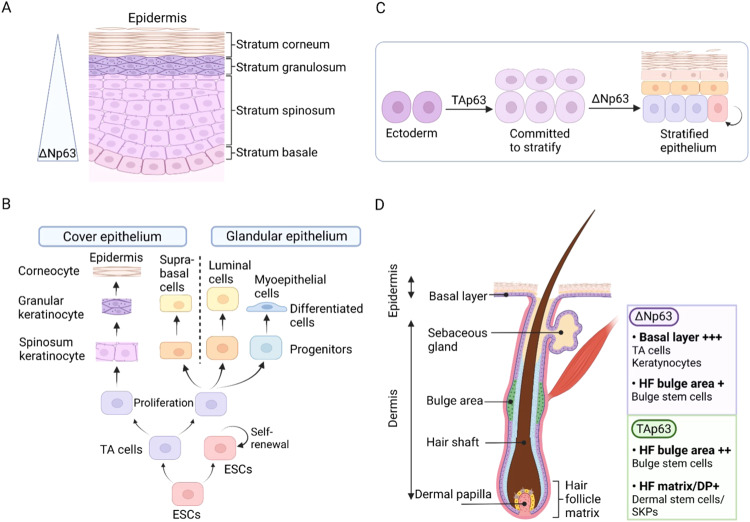
p63 expression in the development of epidermis. **A** Schematic diagram of epidermal structure. The expression of p63, especially ΔNp63 isoform, is higher in basal cells and decreases during the basal-to-suprabasal transition. **B** The ESC differentiation program initiates within the basal layer and generates non-stem-committed TA cells, which give rise to the progenitor population, whose progeny finally differentiates into multiple epithelium lineages. **C** The specific expression of TAp63 and ΔNp63 in different phases during epidermal development. **D** A cross-section of the hair follicle (HF) and the surrounding epidermis, showing differential expression of p63 isoforms in various HF stem cells subsets.

The role of ESCs in the homeostatic maintenance of the skin and in wound repair has been well recognized. Skin epithelium, also known as epidermis, is a stratified squamous epithelium (Fig. 2A). Its proliferative basal compartment consists of ESCs and their progeny, the daughter transient amplifying (TA) cells [49]. ESCs have a strong ability to self-renew to supplement senescent keratinocytes and help to promote healing. ESCs divide asymmetrically to generate a stem and a TA cell. The latter withdraws from the cell cycle and detaches from the basement membrane to begin terminal differentiation (Fig. 2B). This process occurs concomitantly with the movement of the differentiating keratinocyte toward the surface of the skin. p63 is enriched in the basal layer of the epidermis and serves as a specific marker of ESCs, in which it assists in regulating proliferation and differentiation [20, 50]. p63 expression, especially ΔNp63, is reduced at both protein and mRNA levels during the basal-to-suprabasal transition in epidermis [51] (Fig. 2A). Furthermore, p63 expression induces hyperproliferation of ESCs, whereas, downregulation of p63 is needed for terminal differentiation to take place [52]. Epidermal progenitors with depleted p63 have impaired self-renewal potential, which could render them incapable of perpetuating [53]. In keratinocytes (precursor cell derived from ESCs), p63 expression is responsible for cell adhesion, inhibiting apoptosis, and upholding the integrity of the epidermal tissue [54].

Mice lacking ΔNp63 display a dramatic loss of keratinocytes and exhibit single-layered surface epithelium instead of a fully stratified epidermis, perhaps, this is due to the early onset of excessive cell proliferation decline of all stem and TA cells [55, 56]. Ablating TAp63 in the germline (TAp63^-/-^ mice) or in keratin 14 (K14)-expressing cells in the basal layer of the epidermis (TAp63^fl/fl^; K14Cre^+^ animals) leads to impaired proliferation, accelerated cellular aging, and genomic instability in these precursor cells. Moreover, the mice experience premature aging, manifesting blisters formation, skin ulcerations, and impaired hair morphogenesis [57, 58]. The TAp63 isoforms are the first to be expressed during embryonic epidermal development, during which they are necessary for the commitment to epithelial stratification. During later stages of epidermal development, a switch to ΔNp63 isoforms is then required to counterbalance the activity of the TAp63 proteins, thereby allowing cells to respond to terminal differentiation cues [36] (Fig. 2C).

### Skin appendages

In both vertebrates and non-vertebrates, p63 plays a crucial role in the development of epithelial appendages such as hair follicles and many exocrine glands [59, 60]. Both of mice and zebrafish lacking p63 result in failure to develop epithelial appendages and other structures that arise from epithelial-mesenchymal interactions [60].

### Hair follicle

Distinct populations of stem cells located in the interfollicular epidermis and various niches of the hair follicle (HF) are responsible for maintaining the epithelium. Upon injury, the progenies of all stem cells are capable of leaving their respective niches and contributing to the wound healing process known as re-epithelialization [61–64]. In normal human HFs, p63 expression is principally restricted to cells with high proliferative potential, named HF stem cells (HFSCs) [65] (Fig. 2D). It’s also a potential indicator of interfollicular epidermal stem cells (IESCs) [65], while it is absent in cells that are undergoing terminal differentiation [56, 62–64]. During its growth cycle, the HF appears to recapitulate part of its development during embryogenesis, in which p63 is involved [66]. A role for p63 in hair formation has also been well established, as p63-null mice fail to form HF [67, 68]. Furthermore, ΔNp63 is enriched in the developing of hair placode, however, its expression is restricted to the outer root sheath, the matrix cells, and the stem cells of the HF bulge in mature hair. As reported, mice with mutant ΔNp63α exhibited a missing HFSCs niche and further overall cycling defects [69].

### Mammary gland

**Fig. 3:**
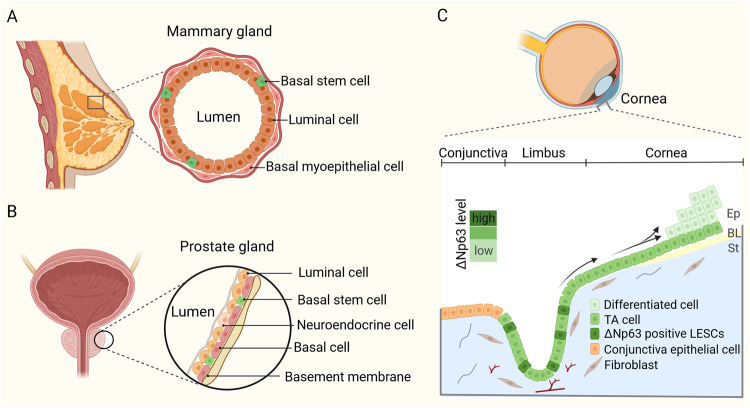
Stem cell in glandular and stratified epithelia. **A** A schematic model depicting the structure of the mammary gland epithelium, composed by basal stem cells, basal myoepithelial cells, and luminal cells. **B** The model shows the distribution of basal stem cells, luminal cells, and neuroendocrine cells in the prostate gland epithelium. **C** Schematic representation of the cornea. LESCs produce daughter TA cells that migrate towards the apical layer of the central cornea, and, eventually, become terminally differentiated (arrowed). ΔNp63 is the dominant p63 isoform expressed in LESCs and its expression decreases from limbus to the central cornea. Ep epithelium, BL Bowman’s layer, St stroma.

The mammary gland consists of a bilayered epithelium that is composed by an inner luminal layer of secretory epithelial cells, and an outer basal compartment of myoepithelial cells, including rare stem cells [70] (Fig. 3A). During embryonic development, sustained p63 expression in multipotent progenitors facilitates the differentiation of unipotent basal cell lineage in mammary gland [71]. TP63 null mice genetically deleted for all the isoforms, display complete lack of mammary glands [20]. Similarly, humans harboring germline p63 mutations exhibit mammary hypoplasia [72]. Subsequent studies indicated that ΔNp63 is the predominant variant governing mammary glands morphogenesis. Romano and collaborators reported that selective genetic ablation of ΔNp63 recapitulates the mammary gland defective phenotype observed in the p63 global knockout mice [73]. In the adult mammary gland, ΔNp63 and TAp63 exhibit segregated expression in mammary stem cells and progenitor cells [74, 75]. ΔNp63, especially the ΔNp63α isoform, is mainly expressed in the basal cell layer that is composed of myoepithelial and stem/progenitor cells, while its expression is maintained very low in luminal cells [76, 77]. TAp63 protein is instead expressed in luminal cells [78]. The expression of ΔNp63 in mammary progenitor cells serves to maintain their undifferentiated state and quiescence and therefore to promote stem cell activity [31, 74, 79–81]. In addition, it has been reported that ΔNp63 can reprogram adult luminal into basal cells by promoting an intermediate multipotent-like state, thus supporting the concept of reversible plasticity in epithelial cell fate [71, 82]. ΔNp63 is expressed in the adult mammary gland during all the physiological stages, virgin, pregnant, lactation, and post-lactation involution states [75]. On the other hand, the mammary glands of p63^+/^^−^ mice appear normal in the virgin, pregnant and lactating states.

### Prostate gland

Normal prostate epithelium contains three major epithelial cell types, basal cells (including stem and TA cells), luminal cells, and neuroendocrine cells [83] (Fig. 3B). In the adult, prostate stem cells can give rise to all three lineages of prostate epithelial cells [84]. p63 has been shown to play an essential role in normal stem cell function during prostate development. At birth, mice lacking p63 manifest prostate bud agenesis and urothelial abnormalities [4, 45]. Prostaspheres derived from p63-expressing basal cells efficiently form organoids expressing the differentiation marker cytokeratin 18 (CK18) and a functional androgen receptor [85]. The prostate agenesis indicates that p63 is required for the formation of prostate stem cells residing in the basal layer of the primitive urogenital sinus epithelium. In the absence of p63, the epithelial lining of the urethra indeed shows lack of stratification and complete absence of basal cells [4]. p63-null blastocyst complementation experiments have demonstrated that p63-positive basal cells are necessary for the development of p63-negative luminal cells [86–88]. Within the prostate epithelium, ΔNp63 is selectively expressed in the basal cell layer, while it is absent in the luminal and neuroendocrine cells [89]. In an in vitro study of prostate cell spheroid, p63-expressing cells are located in the outer layer of the spheres and exhibit self-renewal potential, while cells located in the center display a differentiated phenotype [30]. In addition, another in vitro prostasphere formation assay indicated that inhibiting p63 expression reduces prostate stem progenitor cells and promotes differentiation [90].

### Corneal epithelium

The self-renewing properties of the corneal epithelium are closely related to the integrity and function of the cornea [91, 92], which is closed to stem cells that reside in the basal epithelial layers of the limbal epithelium, called limbal epithelial stem cells (LESCs) [93]. The limbus is a narrow ring of tissue located between the cornea and conjunctive border, among which LESCs give rise to progenitor cells that move toward the corneal center, through the suprabasal layers of the corneal epithelium (Fig. 3C). Under normal condition, the renewal of the corneal epithelium is carried out by LESCs which express higher p63 levels than peripheral or central corneal epithelium cells [94]. The corneal epithelium of p63-null mice fails to maintain continuous development beyond ectodermal stage [95]. Heterozygous mutation in the TP63 gene is also responsible for LESCs failure and results in drastic degeneration of corneal epithelial tissues [96]. ΔNp63α is the predominantly expressed isoform in LESC and in limbal basal cells [97–99], but not in cells that cover the central corneal surface [96] (Fig. 3C). The expression of the ΔNp63α isoform allows LESCs to produce TA cells, which are responsible for the generation and maintenance of the basal layer of the corneal epithelium [96]. Moreover, ΔNp63α is necessary for the maintenance of the proliferative [100] and migration potential of LESCs during corneal wound healing [101]. In the absence of ΔNp63, corneal epithelium renewal and terminal differentiation are impaired. However, an in vitro investigation demonstrated that TAp63 and ΔNp63 expression appears to be specifically located in the limbus, while it and gradually decreases in the cornea, especially disappearing in the central cornea [102]. Furthermore, suppression of each of them affects the proliferation of limbal keratinocytes [102]. Besides, only TAp63 but not ΔNp63 acts in a way that promotes cell differentiation [102].

### Thymus

Thymic epithelial precursor cells derive from the endoderm of the third pharyngeal pouch [103] and appear at E12 along with high levels of p63 expression [29]. p63 is required for maintaining the proliferative potential of stem cells in the thymus epithelium and is indispensable for thymus development and homeostasis. A study focusing on the impact of lineage-restricted p63 knockout (ko) in TECs on pre- and postnatal mice clearly defined that p63TECko mice present severe thymic hypoplasia, which is characterized by a lack in well-defined segregation into medullary and cortical compartments [104]. Interestingly, the skin of p63TECko mice shows continuous epidermis but no hair by 2 months of age [104]. Controversially, another study determined that thymus hypoplasia in p63-null embryo is attributed to decreased proliferative potential and increased apoptosis of TESCs, but p63 deficiency did not alter commitment and functional maturation of TESCs [29]. In an in vitro study, TESCs clones expressing p63 appear small and immature, while p63 shRNA expressing clones are large and present increased expression of the terminal differentiation markers after two-week culture [29], which means the symmetric expansion of TA cells [45]. In the developing of thymus, some abnormalities result from epithelial failure due to the absence of TESCs, which is attributed to the action of ΔNp63 [32]. Correspondingly, ΔNp63 isoform is the most abundant in both cortical and medullary TESCs [29, 105].

### Lung

Club cells are regional progenitor cells involved in the repair of the bronchiolar epithelium in response to lung damage. They give rise to epithelial alveolar type 2 cells (AT2s) and alveolar type 1 cells (AT1s) during the repair process. In addition, the distal airway stem/progenitor cells (DASCs) expressing p63 as well as keratin-5 (K5) are located in the airway basal epithelium and are responsible for lung regeneration after lung injury [106, 107]. In addition, induced p63^+^/KRT5^+^ lung epithelial cells can give rise to alveoli, indicating the stemness potential [108, 109]. Upon mild-to-moderate pulmonary injury, surviving AT2s self-renew and differentiate into oxygen-exchanging AT1 population to carry out functionally beneficial tissue regeneration [110]. Abnormal epithelial remodeling follows severe lung injury, such as the one caused by pandemic H1N1 influenza and COVID-19, which has been shown to progressively induce the proliferation of p63-expressing epithelial cells [111]. Recently, a study focusing on acute lung injury (ALI) demonstrated that active p63 expression promotes proliferation and inhibits apoptosis in epithelial progenitor cells [112]. p63-positive cells arising from the lung primordium are primary multipotent progenitors of airway and alveolar lineages. These cells, located in intrapulmonary airway, maintain immature to adulthood in bronchi, where there is an important reservoir for establishing rare cell population that responds to H1N1 infection [108]. An in vitro investigation indicated that knockdown of p63 affects cellular morphology, proliferation, migration ability and results in impaired wound healing response and premature senescence in the airway epithelium [113]. In particular, ΔNp63 is the principal isoform in human lung and exclusively enriched in the basal cells of the bronchial epithelium [113, 114]. Weiner and colleagues uncovered that ΔNp63 restricts the plasticity of intrapulmonary DASCs by maintaining histone modifications at key gene loci [115]. Accordingly, DASCs undergo either airway or alveolar differentiation following loss of ΔNp63. An age-associated decrease of TAp63 was observed in monkey. In contrast, ΔNp63 expression showed a higher level, which triggers the migration of club cell (the second stem cell population in the airway epithelium) [116].

## Signaling involved in p63-mediated stemness regulation

p63 regulates a wide range of target genes that are important for maintaining stemness in both physiological and pathological conditions. Some of them are discussed in the paragraphs below.

### The Notch pathway

**Fig. 4:**
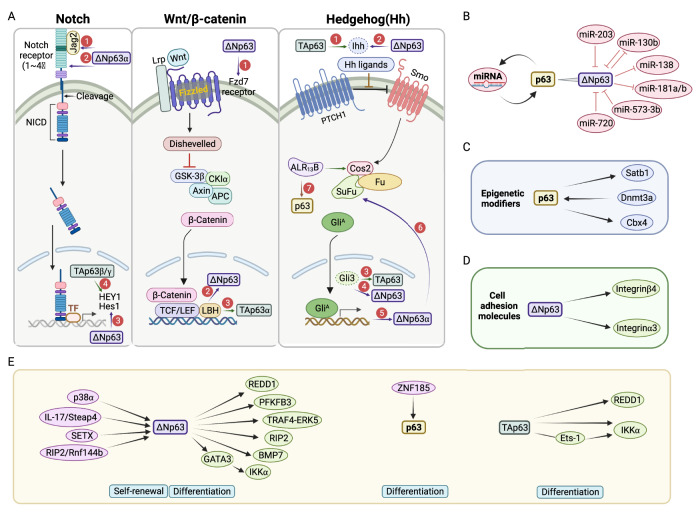
The signaling pathways involved in p63-mediated stemness regulation. **A** The Notch (left), Wnt/β-catenin (middle), and Hh (right) pathway components modulated by p63 isoforms during ESCs’ stemness regulation. All the encircled numbers indicate p63 isoform contribution by transcription regulation. **B** The reciprocal miRNA-p63 interactions are a vital factor in modulating p63 function, which is extensively involved in the development of the skin epidermis, including epithelial cell proliferation, differentiation, and senescence. **C** p63 regulates the stemness of ESCs through the signal molecules associated with epigenetic modifications. **D** ΔNp63 controls the stemness of ESCs by regulating cell adhesion molecules. **E** Other signaling involved in p63-mediated modulation of ESCs’ stemness. As shown, ΔNp63 is the most studied isoform and it is involved in various cell mechanisms to achieve self-renewal and differentiation control of ESCs.

A role for Notch signaling in stem cell preservation and cell fate determination has been described. Notch activation is able to promote self-renewal of stem cells, proliferation of progenitor cells, and commitment to specific cell lineages [117]. Notch proteins are cell-surface receptors (Notch1~4) that transduce signals upon interaction with transmembrane ligands such as Delta and Jagged (Jag), expressed on adjacent cells. They comprise an extracellular (NECD), a transmembrane (TM), and an intracellular (NICD) domain. In the canonical Notch signaling pathway, after three cleavages, the NICD directly translocates into the nucleus, where it mediates signaling through conversion of the DNA-binding protein from a repressor to an activator of transcription for the activation of target gene expression [118] (Fig. 4A, left panel).

Increased expression of Notch2 and Notch3 but decreased levels of Hes1 (Hairy and Enhancer of Split 1) and Notch1 were observed in the dorsal skin of mice lacking ΔNp63 at E15.5, accompanied by diminished renewal capacity and an altered differentiation fate [73] (Fig. 4B, left panel). During thymic development, Jag2 displays high expression levels in the presence of ΔNp63, by which Jag2 is involved in the maintenance of TECs’ stemness [32]. In addition, in mouse keratinocytes, ΔNp63α blocks Notch1-dependent growth arrest and differentiation by inhibiting p21 expression as well as maintaining integrin expression [119]. On the other hand, TAp63β/γ activates the transcription of late differentiation genes through the cooperation with Notch signaling. In particular, it upregulates HEY1(Hes-related family bHLH transcription factor with YRPW motif 1) in human keratinocytes [120].

p63 and Notch also play antagonistic roles in controlling epithelial cell fates. In both mouse and human keratinocytes, p63 expression is inhibited by Notch1, while in contrast, upregulation of p63 oppositely regulates Notch target genes, mainly Hes1, and restricts the capability of Notch1 for inhibiting cell proliferation and promoting differentiation [51]. The Notch signaling pathway is activated during ectoderm specification, while its inactivation is necessary for promoting ΔNp63 expression and epidermal keratinocyte lineage commitment [121]. As enriched ΔNp63 expression indeed confers a basal phenotype in purified luminal cells, while Notch activation specifies a luminal cell fate [82]. Sierra Kent and colleagues demonstrated that ΔNp63α inhibits cell-cycle progression and promotes cellular quiescence of mammary epithelial cells, predominantly through Notch3 regulation, thus providing novel mechanistic insights into the maintenance of mammary cellular quiescence by the p63/Notch axis [122]. In summary, the reciprocal antagonistic interactions of p63 isoforms and Notch family members determine the proper output of stemness regulation.

### The WNT/β-catenin pathway

WNT/β-catenin pathway is a highly conserved pathway involved in the maintenance of the progenitor cell population in the skin, limbus, intestine, mammary gland, and other epithelial tissues [123–126]. It’s initiated by the interaction of the WNT ligands with two cell-surface receptors Frizzled (Fzd) and an Lrp (Fig. 4A, middle panel). Then, induced Disheveled (DVL) signaling cascade inhibits the β-catenin destruction complex in the cytoplasm, where β-catenin is constantly phosphorylated by the glycogen synthase kinase-3β (GSK-3β) and, subsequently, rapidly ubiquitinated and targeted for proteasomal degradation. Finally, activated β-catenin translocates into the nucleus and regulates the expression of numerous target genes [127].

An investigation in normal mammary stem cell (MaSC) demonstrateed that ΔNp63 maintains their self-renewal ability through directly upregulating the expression of Fzd7 [31]. In turn, the WNT/β-catenin signaling could also be the upstream regulator of ΔNp63, because of the presence of a putative β-catenin-RE within its promoter region. Limb-Bud and Heart *(*LBH), a transcription co-factor in the WNT pathway, upregulates the transcription of ΔNp63α but downregulates TAp63α expression. By this way, LBH enhances the replicative potential as well as stemness of mammary basal ESCs and inhibits the transcription of luminal differentiation genes, such as Esr1/ERα (estrogen receptor α) [81].

### The Hedgehog signaling pathway

The Hedgehog (Hh) signaling is triggered by the binding of one of the three secreted ligands, Sonic Hedgehog (Shh), Indian Hedgehog (Ihh) or Desert Hedgehog (Dhh) to the Patched1 (PTCH1) receptor. Upon binding, PTCH1 releases its inhibition on Smoothened (SMO), that then transduces the Hh signal across the cell membrane, activating the Glioma-associated oncogene homologs family (GLI1, GLI2, and GLI3) of transcription factors [128].

The Hh signaling pathway is a crucial driver of ESC self-renewal [129]. Reciprocal interactions between p63 and the Hh signaling components regulate quiescence and activation of ESCs (Fig. 4A, right panel). p63 contributes to the stemness phenotype through its ability to bind to the promoters of Hh components, assigning positive and negative regulation to Ihh by TAp63 and ΔNp63 isoforms, respectively [75]. Conversely, Ihh-induced GLI3 upregulates TAp63 expression but reduces the usage of the ΔNp63 promoter, through which the Hh signaling plays an important role in the elaboration and differentiation of mammary progenitors and deceased clonogenicity of MaSCs in vitro [75]. During keratinocyte differentiation, an active Hh signaling pathway is observed in proliferating cells, in which it triggers the expression of ΔNp63α and further activates SUFU transcription. In turn, increased SUFU inhibits GLI-targeted genes, including ΔNp63α, and then restrains proliferation and promotes differentiation of keratinocytes [130]. On the other hand, constitutively activated Hh signaling in palatal epithelium results in the persistence of p63 signaling and p63-targeted genes in medial edge epithelium, which directly enhancing the epithelial progenitor cell-associated genes [131]. In epididymal, ARL_13_B is responsible for epithelium integrity and tissue regeneration. In its absence, the Hh signaling is impaired and basal cell markers, such as p63 are lost [132].

### Noncoding RNAs

The reciprocal microRNA (miRNA)-p63 interaction is a vital factor that modulates p63 function [133], and miRNA plays a crucial role in the modulation of stem cell senescence and proliferation [134–137] (Fig. 4B). miR-203 is a key molecule controlling the proliferative potential and inducing cell-cycle arrest of epithelial precursor cells during epithelial development. Mechanistically, it inhibits p63 activity through targeting human and mouse p63 3’-UTR, especially ΔNp63 [44, 55, 138]. Consistently, increased expression of miR-203 by oleic acid (OA), an unsaturated free fatty acid (FFA), enhances the differentiation of keratinocytes through reducing p63 expression [139]. Besides, the proliferation of human primary keratinocytes in vitro coincides with the increased miR-130b, whose overexpression will induce cell senescence through inhibition of ΔNp63 expression. However, ΔNp63α, in turn, inhibits miR-130b expression in primary keratinocytes, thus promoting their growth [140]. ΔNp63α-inhibited miR-138, miR-181a, miR-181b, and miR-130b participate in the growth-promoting of epidermal proliferating cells and keratinocyte senescence [140]. Furthermore, miR-574-3p and miR-720 function as regulator of keratinocyte differentiation by linking iASPP, an inhibitory member of ASPP (apoptosis stimulating protein of p53) family, and silencing of iASPP in keratinocytes accelerates the differentiation pathway through indirectly increasing protein level of ΔNp63 [141].

Several long noncoding RNAs (lncRNAs) have been implicated in keratinocyte biology, through either the maintenance of the undifferentiated state of the progenitor cells or the induction of epidermal differentiation [142–144]. In addition, lncRNAs are crucially involved in the development and progression of several human epithelial cancers [142, 145–149]. There is emerging evidence that some of the lncRNAs regulating epidermal differentiation are functionally related to p63. For instance, beta1-adjacent-lncRNA (BLNCR) is a p63-regulated lncRNA, whose expression has been associated with the proliferative potential of epidermal stem cells since it undergoes rapid downregulation upon differentiation induction [150]. Fierro and colleagues identified a significant number of annotated lncRNAs, whose expression was differentially regulated in epidermal keratinocytes upon ΔNp63 inactivation [151]. Among the most modulated lncRNAs in p63-depleted cells, they found downregulation of MIR17HG and BCRNY, and upregulation of FTX, MALAT1, and NEAT1. In proliferating keratinocytes, ΔNp63 negatively regulates the expression of MALAT1 and NEAT1 by recruiting the histone deacetylase HDAC1 to their promoter regions. HDAC1 then reduces histone H3 acetylation on the promoter regions of NEAT1 and MALAT1, ultimately leading to their transcriptional repression. Upon induction of differentiation, ∆Np63 downregulation results in enhanced levels of NEAT1, which then controls the expression of epidermal genes to promote keratinocyte differentiation.

### Epigenetic modifiers

ESCs’ stemness properties are also under the control of epigenetic modifications (Fig. 4C). p63 binds to the regulatory region of Satb1 gene, a genome organizer, contributing to epidermal morphogenesis through tissue-specific chromatin organization [152]. As demonstrated, the expression Satb1 in p63 (+/−) mice skin explants partially restores the epidermal phenotype. The endogenous DNA methyltransferase 3 A (DNMT3a) cooperates with p63 to maintain a high level of DNA hydromethylation at enhancer sites in a Tet2-dependent manner, thus preserving the self-renewal potential of human epidermal stem cells (EpSCs) [153]. Cbx4, a component of Polycomb Repressive Complex 1 (PRC1) highly expressed in thymus epithelium, physically interacts with p63 and thereby contributes to the maintenance of stemness in epithelial progenitors [154]. Similarly, Cbx4 is decreased in p63 knockout keratinocytes, and Cbx4 knockout mice exhibit impaired cell proliferation and premature terminal differentiation during epidermis development [155].

### Cell adhesion molecules

Interestingly, p63 has also been reported to participate in the regulation of anoikis by modulating cell adhesion [156], which is essential for progenitor cell maintenance. The interaction between integrins and receptors for growth factors and cytokines creates a complex web of signaling pathways that impact ESCs niche. As depicted in Fig. 4D, deficiency of p63 results in the downregulation of genes associated with cell adhesion, such as ITGB4, which encodes β4 integrin (also known as CD29). ΔNp63α specifically upregulates β4 integrin, leading to cell detachment and anoikis in mammary epithelial cells and keratinocytes. Consistently, β4 integrin partially protects against p63 loss-induced anoikis [156]. Furthermore, the knockdown of p63 (TAp63γ and ΔNp63α) impairs the expression of cell adhesion molecules, such as human ITGA3, which encodes the integrin subunit α3 that combines with integrin β1 to form the α3β1 dimer, by which p63 is thought to facilitate the anchoring of ESCs to their niche [156, 157].

### Other signaling

There are many other signaling molecules that are also involved in p63-mediated regulation of epithelial stem cell stemness (Fig. 4E). Metabolic regulation [158, 159], and reactive oxygen species (ROS) [160] are essential in all biological functions. The epidermis is subjected to significant oxidative stress due to the production of ROS during aerobic metabolism [161, 162]. REDD1 (regulated in development and DNA damage responses 1), a regulator in DNA damage response (DRR), appears to function as a regulator of cellular ROS. p63 expression, especially TAp63γ and ΔNp63α, coincides with that of REDD1 in undifferentiated human primary keratinocytes. The downregulation of anyone of this two isoforms dramatically restricts REDD1 expression and promotes the differentiation program through increased ROS levels, suggesting that the p63-REDD1-ROS axis is responsible for preserving the undifferentiated state [163]. p63 isoforms are also known as vital regulators of energy metabolism. Hamanaka and Mutlu proposed that p63 affects cellular glycolytic rate of keratinocytes by regulating the transcriptional expression of metabolic enzyme phosphofructokinase-2/fructose-2,6-bisphosphatase 3 (PFKFB3), thereby resulting in a proliferation/differentiation switch [164, 165]. Smirnov and colleagues demonstrated that Zinc finger protein 185 (ZNF185) contributes to maintain epithelial integrity and epithelial differentiation potential through the specific binding of p63 to its enhancer region in keratinocytes [166]. In the mouse epidermis, the activation of protein kinase p38α phosphorylates and destabilizes ΔNp63α. Conversely, p38α deficiency stabilizes ΔNp63α and promote keratinocyte colony formation [167]. Steap4 (Six-Transmembrane Epithelial Antigen of Prostate 4)-ΔNp63 axis induced by interleukin-17 (IL-17) is positively related to the TRAF4 (TNF Receptor-Associated Factor 4)-ERK5 axis through p63-mediated TRAF4 expression, by which IL-17 favors keratinocyte proliferation [168]. Gatti et al. found that Senataxin (SETX), an RNA/DNA helicase, acts as a key molecule of skin homeostasis by regulating a series of genes involved in the early step of keratinocyte differentiation program together with ΔNp63 [169]. Additionally, the RING finger E3 ubiquitin ligase PIR2 (also known as Rnf144b) has been identified as a target of ΔNp63α, which in turn induces the degradation of ΔNp63α, impairing keratinocyte proliferation and differentiation [170]. In the mammary epithelium, ΔNp63 activates canonical bone morphogenic proteins (BMPs) signaling by inducing the transcription of BMP7, which then fosters the stem cell phenotype of mammary progenitors [171]. IκB kinase (IKK), one of the three main subunits of NF-κB complex, consists of two catalytic subunits, IKKα and IKKβ, as well as a regulatory subunit, IKKγ. TAp63 acts as the upstream regulator of IKKα through direct transactivation and also indirectly through Ets-1(E26 transformation-specific-1). In contrast, ΔNp63 only indirectly activates IKKα through GATA3 in keratinocytes during epithelial development [172].

## p63 and cancer stem cell in epithelial tumor

**Fig. 5:**
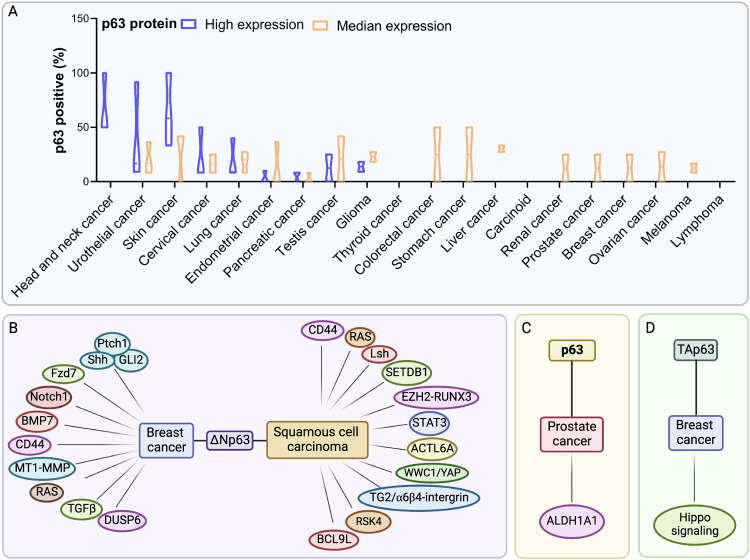
The signaling pathway by which p63 affects CSCs in epithelial cancers. **A** Distinct expression levels of the p63 protein in different tumor tissues (The Human Protein Atlas, https://www.proteinatlas.org/ENSG00000073282-TP63/pathology). **B** The ΔNp63 isoform plays a crucial role in regulating various signaling pathways implicated in CSC stemness regulation, particularly in breast and SCC cancers. **C** p63 regulates ALDH1A1 in the context of prostate cancer, in which p63 affects CSC stemness. **D** TAp63 modulates the expression of specific Hippo pathway components to regulate the stemness of CSCs in breast cancer.

TP63 is rarely mutated in human cancers but its expression and activity are often increased [173–175] (Fig. 5A). The human TP63 gene exhibits increased expression in 88% squamous carcinomas and 42% of large cell carcinomas and lung adenocarcinomas [176]. Among p63 isoforms, ΔNp63 is the predominant expressed isoform in various epithelial tumors and its alterations positively correlate with increased metastatic potential and therapy resistance [174, 177, 178]. Emerging evidence suggests that several tumors are maintained by tumor-initiating cells (TICs), also referred as cancer stem cells (CSCs), possessing stem-like properties [179, 180]. CSCs are considered as the cellular “seeds” of tumor recurrence, metastasis and therapy resistance [181–184]. Like in normal tissues, self-renewal and differentiation of rare CSCs are essential and responsible for the growth of the tumor mass [185, 186]. Growing evidence suggests that abnormal signaling within pathways that regulate the stemness properties of normal stem cells may contribute to foster survival of CSCs. Most importantly, increasing evidence is pointing at the regulation of CSCs by p63 in various epithelial cancers [87, 174].

ΔNp63 is enriched in breast cancer CSCs and promotes their colony formation ability [31] (Fig. 5B). Memmi and colleagues reported that ΔNp63 is preferentially expressed in CSCs from transgenic mice with mammary gland targeted ErbB2 (MMTV-ErB2) tumors and is required to maintain their self-renewal potential. Mechanistically, ΔNp63 directly binds to the gene regulatory regions of Shh, Gli2, and Ptch1 genes, regulating their expression and ultimately contributing to pathway activation [187]. Additionally, p63 inactivation in ErbB2 progenitors reduces the transcription of genes for encoding components of the Shh signaling, to which mainly ΔNp63 isoform contributes, thus attenuating mammary CSC self-renewal [187]. Similar to the normal counterparts, the tumor-initiating activity of CSCs of basal breast cancer is governed by ΔNp63-dependent transcription of Fzd7 [31]. A clinical study showed that ΔNp63 was enriched in a cell subpopulation from triple negative breast cancer patients. Furthermore, ΔNp63α positively correlates with Notch1 expression by directly binding to its promoter, and increases the fraction of CSC-like cells in a luminal breast cancer cell line [188]. The interaction between ΔNp63 and BMPs, especially BMP7, is able to commit human breast cancer cells to a stem-like phenotype [171]. The ΔNp63 isoform stimulates the transcription of the gene that encodes the cell-surface antigen CD44, a marker associated with cancer stem cells in breast, colorectal, and squamous cell carcinomas [189, 190]. Albeit at levels lower than in normal prostate, p63 expression is essential for regulating stem cell functions of neoplastic prostate epithelial cells. Cytoplastic aberrance of p63 is associated with high ALDH1A1(Aldehyde Dehydrogenase 1 Family, Member A1) expression, thus leading to increased cell proliferation rate of prostate CSCs [191] (Fig. 5C). Su and colleagues demonstrated that TAp63 is the main regulator of the transition from mammary cells to TICs (Fig. 5D). In the absence of TAp63, mammary epithelial cells (MECs) unleash stemness potential by expressing MaSCs signatures as well as by activating the Hippo pathway. Therefore, TAp63 null mice develop aggressive metastasis [192].

In the skin, ΔNp63α acts as an oncogene, capable of inhibiting oncogene-induced senescence and promoting development of squamous cell carcinoma (SCC) (Fig. 5B). ΔNp63α cooperates with oncogenic RAS to promote proliferation and to maintain the KRT15-positive stem cell population in order to drive tumorigenesis in vivo [193]. The expression of Lsh, a chromatin-remodeling protein is regulated by ΔNp63α during senescence bypass and tumor initiation, suggesting that p63 plays a role in epigenetic regulation of stemness [193]. Enhancer zest homolog 2 (EZH2) and SET domain bifurcated 1 (SETDB1) are two well-known oncogenic methyltransferases that are highly expressed in stem-like SSC sub-populations. SETDB1 cooperates with ΔNp63α to regulate the SCC stem cell phenotype. In addition, EZH2-activated RUNX3 acts as an inhibitor of both SETD1 and ΔNp63α [194]. ΔNp63 is often overexpressed in SCCs, in which it acts as a master regulator of CSCs maintenance via its functional connection with signal transducer and activator of transcription 3 (STAT3), assigning STAT3 induces ΔNp63 expression through binding to its promoter [195–197]. The gene encoding the chromatin-modifying protein that ACTL6A (Actin-like protein 6 A) is coamplified with *p63* locus in HNSCC and induces a CSC phenotype characterized by undifferentiated regenerative proliferation. They also found ACTL6A/p63 complex is a direct transcription suppressor of Hippo regulator WWC1 and thus promotes YAP-mediated tumorigenesis [198]. Additionally, transglutaminase 2 (TG2) is an effector required for CSC survival in epidermal SCC. The interaction between TG2 and α6/β4-integrin is able to induce YAP-dependent ΔNp63α stabilization and therefore leads to enhanced tumor stem cell properties [199]. The Ribosomal S6 protein kinase 4 (RSK4) is one of the direct downstream targets of ΔNp63α in CSC maintaining in esophageal squamous cell carcinoma. As found, ΔNp63α directly transactivates RSK4 expression, thereby inhibiting radiosensitivity through GSK-3β/β-catenin axis [200]. Additionally, ΔNp63 regulates the self-renewal and maintenance of LESCs, which are indispensable for lung adenocarcinoma (LUAD) and squamous cell carcinoma (LUSC) initiation as well as progression through the regulation of a common landscape of enhancer-associated cell identity genes, such as BCL9L (B-cell CLL/lymphoma 9-like) [201].

Altered expression of p63 in tumors correlates with aberrant expression of p53 or with the presence of *TP53* mutants, indicating that the p63-p53 axis is relevant to tumorigenesis. Several p53 mutant proteins can interact with p63 through the OD, resulting in the inhibition of the transcriptional activity of the TAp63 variants and, subsequently, of their tumor suppressive behavior [2, 202]. Other p53 mutations occurring in the DBD render this domain very unstable, thus resulting in p63α binding via a co-aggregation mechanism mediated by the TIDs of the α-isoforms [203]. Inactivation of p63 by p53 mutants has been associated with the ability of mutant p53 proteins to promote tumor growth, chemoresistance, and metastasis [204]. Mechanistically, during tumor progression, tumor growth factor-β (TGF-β), acting in concert with oncogenic RAS, induces the formation of a mutant-p53/p63/SMADs ternary protein complex, in which the SMADs are essential platforms for the complex to assembly [202]. As a result of this interplay, the anti-metastatic properties of TAp63 are inhibited, allowing cancer cells to acquire pro-invasive and migratory capabilities. Mutant p53 proteins was also found to recruit p63 to gene promoters. In this context, p63 serves as a molecular chaperone through which p53 mutants aberrantly reprogram the cancer cell transcriptome and facilitate tumor invasion [205]. Zhang and colleagues reported that mutant p53 is capable of antagonizing p63-mediated tumor suppression in an in vivo model of T lymphoblastic lymphomas (T-ALL) [206]. In particular, mutant p53 contributes to the pathogenesis of T-ALL by collaborating with NOTCH1 to hijack p63-mediated transcription. Other studies have highlighted the ability of some p53 mutants to potentiate ΔNp63 function via protein-protein interaction [207].

Growing evidence has implicated p63 as a critical factor in driving the metastatic cascade (Fig. 5B). ΔNp63 acts as a vital regulator of quasi-mesenchymal CSCs which drive the metastasis events, and is required for the post-extravasation proliferation of CSCs in human breast cancer [208]. In the early stage, ΔNp63α is responsible for local invasion through collaborating with the membrane-type 1 (MT1)-matrix metalloproteinase (MMP) in basal-like BC, assigning to ΔNp63α the role in inhibiting the transcription of MT1-MMP [209]. One of the main traits of CSCs is the epithelial-mesenchymal transition process. Activation of oncogenic RAS or the TGF-β signaling in breast cancer cells stimulates ΔNp63 transcriptional activity and then induces the transcription of the dual-specificity phosphatase 6 (DUSP6) gene, whose expression is mainly restricted to stem cells [210]. As a result, DUSP6 empowers ΔNp63 and promotes metastatic behaviors in breast cancer cells [211]. In prostate adenocarcinomas, ΔNp63 is associated with the onset of basal-like cancer stem cell population but not with metastasis, as none of the tumors expressing p63 showed bone metastasis [212].

## Conclusions

p63 is a p53 family protein responsible for morphogenesis and postnatal regeneration of epithelial tissues [213]. TAp63 and ΔNp63 isoforms, expressed in a variety of epithelial tissues and in epithelium-derived tumors, affect the transcriptions of genes involved in different mechanisms of stemness regulation. In this review, we outline the contribution of TAp63 and ΔNp63 isoforms to stemness in epithelial tissues. We also report about their involvement in the regulation of multiple signaling pathways, in both physiological and pathological conditions. In general, by sharing the ability of p53 to induce cell-cycle arrest and apoptosis, TAp63 often acts as a tumor suppressor [23, 214]. In contrast, ΔNp63 mainly works as an oncogene through orchestrating several tumor-promoting pathways, thus being suggested as a marker of CSCs [215, 216]. Therefore, the balance between TAp63 and ΔNp63 appears to modulate cell fate decisions. In either normal ESCs or CSCs, our work clearly shows that p63 participates in multiple pathways related to the regulation of self-renewal and functional differentiation. Collectively, p63 isoforms play an indispensable role in regulating normal ESCs and CSCs stemness for epithelium regeneration and tumorigenesis, respectively. Further investigations on p63 upstream signaling and affected targets would allow to acquire thorough insights into the network centered on this pivotal transcription factor, finally contributing to the achievement of a deeper knowledge of stemness mechanisms, aimed at the development of new therapeutic strategies.
